# Development of a radiomics-based model for diagnosis of multiple system atrophy using multimodal MRI

**DOI:** 10.3389/fneur.2025.1650350

**Published:** 2025-09-08

**Authors:** Zhichao Li, Wei Zhang, Ran Yang, Dong Chen, Xin Li, Kun Wang, Lei Cheng, Heng Yang, Yili Deng

**Affiliations:** ^1^Department of Radiology, Chongqing Western Hospital, Chongqing, China; ^2^Department of Radiology, Second People's Hospital of Jiulongpo District, Chongqing, China; ^3^Department of Internal Medicine, Second People's Hospital of Jiulongpo District, Chongqing, China

**Keywords:** radiomics, magnetic resonance imaging (MRI), diagnostic model, machine learning, multiple system atrophy, neurodegenerative disorders

## Abstract

**Introduction:**

Multiple system atrophy (MSA) is a rapidly progressive neuro-degenerative disorder characterized by autonomic dysfunction, levodopa- unresponsive parkinsonism, cerebellar ataxia, and corticospinal tract involvement. Early diagnosis remains challenging due to overlapping clinical manifestations and the absence of reliable biomarkers. This study aimed to develop a radiomics-based diagnostic model using multimodal MRI to improve MSA detection.

**Methods:**

A retrospective cohort of 62 clinically probable MSA patients (per the 2022 Movement Disorder Society criteria), and 73 matched healthy controls underwent 3.0-T MRI (T1WI, T2WI, FLAIR, DWI). Seven brain regions (bilateral cerebellar hemispheres, middle cerebellar peduncles, putamen, and pons) were manually segmented. A total of 1,502 radiomics features were extracted per region, using PyRadiomics (IBSI-compliant). Features with an intraclass correlation coefficient (ICC) ≥ 0.75 were retained, and the least absolute shrinkage and selection operator (LASSO) regression identified the top discriminative features to construct region-specific radiomics scores (Rad-scores). A logistic regression (LR) model integrated Rad-scores from all regions. Model performance was evaluated via precision, recall, and F1-score in training, testing, and validation cohorts (split ratio 6:2:2), and compared with visual assessments by two radiologists.

**Results:**

The LR model achieved high performance: accuracy was 0.98 in the training cohort, 0.97 in the testing cohort, and 0.95 in the validation cohort. Notably, classification precision for MSA reached 1.0 (indicating no false positives) across all cohorts. SHapley Additive exPlanations (SHAP) analysis revealed that the left putamen Rad-score as the most influential predictor. The model significantly outperformed radiologists' visual assessments (radiologist AUCs: 0.559 and 0.535; *P* < 0.001). Asymmetry was observed, with left-hemisphere structures (putamen/cerebellar) exhibiting greater diagnostic contributions.

**Conclusion:**

Multimodal MRI radiomics accurately differentiates MSA from healthy controls, even in the absence of conventional MRI markers. The Rad-score model demonstrates high sensitivity (89% recall in the validation cohort) and perfect specificity (100% precision), providing a clinically actionable tool for early MSA diagnosis.

## Introduction

Multiple system atrophy (MSA) is a neurodegenerative disorder of unknown etiology and insidious onset, characterized primarily by autonomic dysfunction, poorly levodopa-responsive parkinsonism, cerebellar ataxia, and corticospinal tract dysfunction (1). MSA diagnosis remains challenging due to overlapping clinical manifestations with other neurodegenerative diseases and the lack of reliable biomarkers (2, 3). Epidemiological studies indicate that MSA progresses rapidly with shortened survival, underscoring the critical importance of early diagnosis for symptom management, prognosis evaluation, precision therapy development, and drug discovery (4).

Historically, MSA diagnosis relied on clinical symptoms, signs, and neuroimaging findings (5, 6). Although neuropathological examination remains the gold standard, biopsy-associated risks and patient reluctance limit its utility. Clinical diagnosis alone faces limitations due to phenotypic heterogeneity and symptom overlap across neurodegenerative disorders. Consequently, neuroimaging has been incorporated as supportive evidence in diagnostic criteria (7, 8). Previous studies identified key MRI features: in the MSA-P subtype: Hypointensity in the putamen on T2-weighted imaging (T2WI) and susceptibility-weighted imaging (SWI), with hyperintensity on T2^*^ sequences (9); in the MSA-C subtype: the “hot cross bun sign” (pontine cruciform hyperintensity on T2WI/FLAIR) and middle cerebellar peduncle (MCP) hyperintensity. The “hot cross bun sign” exhibits 99% specificity and 45% sensitivity in differentiating MSA-C from spinocerebellar ataxias, while MCP hyperintensity shows 99% specificity and 68% sensitivity (10). The grading of pontine “hot cross bun sign” (11) correlates positively with cerebellar ataxia severity in MSA-C. These characteristic MRI markers aid in distinguishing MSA from Parkinson's disease (PD), progressive supranuclear palsy (PSP), and sporadic late-onset ataxia, though sensitivity in early-stage disease remains suboptimal (12). While PET-CT and SPECT offer diagnostic value, high cost and radiation exposure hinder widespread clinical adoption (13, 14). Transcranial sonography further suffers from limited sensitivity and specificity (15).

In 2022, the International Movement Disorder Society updated diagnostic criteria, stratifying MSA into four tiers: Neuropathologically established, Clinically established, Clinically probable, Possible prodromal MSA (6). The same year, China released its expert consensus, aligning with international standards while incorporating regional evidence (16). This consensus explicitly mandates multimodal MRI—including T1 (axial/sagittal), T2, ADC, SWI, and T2 FLAIR sequences—as essential for diagnosis, differential evaluation, and disease monitoring. It emphasizes that precise diagnosis requires integrating clinical, imaging, and laboratory data, highlighting the need for novel methods to enhance diagnostic accuracy (12).

Despite these advances, there remains a pressing need for more sensitive and objective imaging diagnostic model. This study aims to develop optimal diagnostic model for MSA based on radiomics features derived from multimodal MRI, providing a novel and precise diagnostic tool for clinical practice.

## Materials and methods

### Subjects

This retrospective study analyzed image data from 69 patients with multiple system atrophy (MSA) admitted to the Second People's Hospital of JiuLongPo district between October 2022 and June 2024. All patients underwent brain MRI prior to admission. Patients were included if they met the following criteria: (1) Diagnosis of clinically probable MSA according to the 2022 International Movement Disorder Society (MDS) diagnostic criteria (6); (2) Completion of standardized brain MRI protocols, including T1-weighted imaging (T1WI), T2-weighted imaging (T2WI), fluid-attenuated inversion recovery (FLAIR), and diffusion-weighted imaging (DWI); (3) No treatments potentially affecting MRI findings within 3 months before enrollment. Patients were excluded for: (1) Comorbid neurological disorders (e.g., stroke, other neurodegenerative diseases); (2) Use of neuroactive medications within 3 months; (3) History of neurosurgery altering brain structure; (4) Incomplete MRI sequences (missing T1WI, T2WI, or T2-FLAIR) or significant artifacts compromising image quality. Based on these criteria, 7 patients were excluded (1 with a history of cerebral hemorrhage, 4 with cerebral infarction lesions, 2 with severe MRI artifacts). Ultimately, 62 patients with clinically probable MSA were included. Healthy normal controls (*n* = 73) were selected from individuals undergoing routine brain MRI at the hospital's health examination center during the same period. Controls were matched to patients for age, sex, and educational level. Exclusion criteria for controls: Family history of neurological disorders; Use of centrally acting medications; MRI evidence of asymptomatic cerebral infarction or white matter hyperintensities.

This retrospective study was approved by the Ethics Committee of the Second People's Hospital of JiuLongPo district. Written informed consent was waived in accordance with national ethical guidelines due to the retrospective nature of the research (17).

### MRI acquisition protocol

All participants underwent brain MRI in the supine position using a 3.0-T scanner (Siemens VIDA, Siemens Healthineers, Erlangen, Germany). Imaging was performed with body coil transmission and 20-channel phased-array head/neck coil for signal reception. The standardized protocols included:

1. T1-weighted Imaging (T1WI): Sequence: fast low-angle shot (FLASH), Orientation: Axial, Parameters: TR = 236 ms, TE = 2.46 ms, Slice thickness = 5 mm, FOV = 220 × 220 mm^2^, Matrix = 202 × 288, Averages = 1.
2. T2-weighted Imaging (T2WI): Sequence: turbo spin echo (TSE), Orientation: Axial, Parameters: TR = 1,500 ms, TE = 80 ms, Echo train length = 198, Slice thickness = 5 mm, FOV = 220 × 220 mm^2^, Matrix = 256 × 320, Averages = 1.
3. T2-fluid-attenuated inversion recovery (FLAIR): Sequence: turbo inversion recovery spin echo, Orientation: Axial, Parameters: TR = 9,000 ms, TE = 84 ms, Inversion time (TI) = 2,500 ms, Slice thickness = 5 mm, FOV = 220 × 220 mm^2^, Matrix = 192 × 256, Parallel imaging acceleration factor = 1.
4. Diffusion-weighted Imaging (DWI): Sequence: single-shot echo planar imaging (SS-EPI), Orientation: Axial, Parameters: TR = 4,200 ms, TE = 68 ms, *b*-values = 0 and 1,000 s/mm^2^, Slice thickness = 5 mm, FOV = 220 × 220 mm^2^, Matrix = 116 × 120, Number of diffusion directions = 3.

Imaging coverage extended from the vertex to the foramen magnum, encompassing the entire cerebrum, brainstem, and cerebellum.

### Image processing and feature extraction

All imaging data were exported from the scanner in DICOM format and converted to NIfTI format using MRIcroGL software (v2.1.60; Chris Rorden, University of South Carolina, USA). The resulting NIfTI files were imported into the open-source medical imaging platform 3D Slicer (18) (v5.7.0; Slicer Community, http://www.slicer.org) for subsequent processing.

Segmentation of seven brain regions was independently performed by two certified radiologists (each with more than 10 years of specialized experience): left cerebellar, left middle cerebellar peduncle (MCP), left putamen, pons, right cerebellar, right MCP, and right putamen (6).

The segmentation workflow included: the ROIs of T1WI, T2WI, FLAIR and ADC sequences were manually delineated along the boundaries of the above brain regions, and the volume of interest (VOI) of each brain region was constructed by ROI interpolation (19, 20).

Standardized radiomics feature extraction was performed through a three-stage protocol: (1) Segmented images underwent isotropic resampling to a uniform voxel resolution of 1 mm^3^ using third-order B-spline interpolation to minimize interpolation artifacts; (2) Feature calculation was executed via the open-source Python library PyRadiomics (v3.1.0a2) (21), with all parameters strictly compliant with the Image Biomarker Standardization Initiative (IBSI) guidelines (21) to ensure reproducibility; (3) Four feature classes were extracted, including morphological features from original images to quantify volumetric and shape characteristics (e.g., sphericity, surface area), texture features from original images capturing spatial intensity heterogeneity (e.g., gray-level co-occurrence matrix metrics), frequency- domain features derived from wavelet-transformed images for multiscale frequency component analysis (e.g., Haar wavelet decompositions), and edge-enhanced features generated via Laplacian of Gaussian (LoG) filtering (σ = 1.0–7.0 mm) to accentuate microstructural boundaries and high-frequency details.

Multimodal radiomics feature integration was achieved by concatenating 1,502 radiomics features extracted from each brain region in each sequence.

Mathematical definitions of texture features followed the PyRadiomics documentation (https://pyradiomics.readthedocs.io/en/latest/features.html).

**Figure 1:**
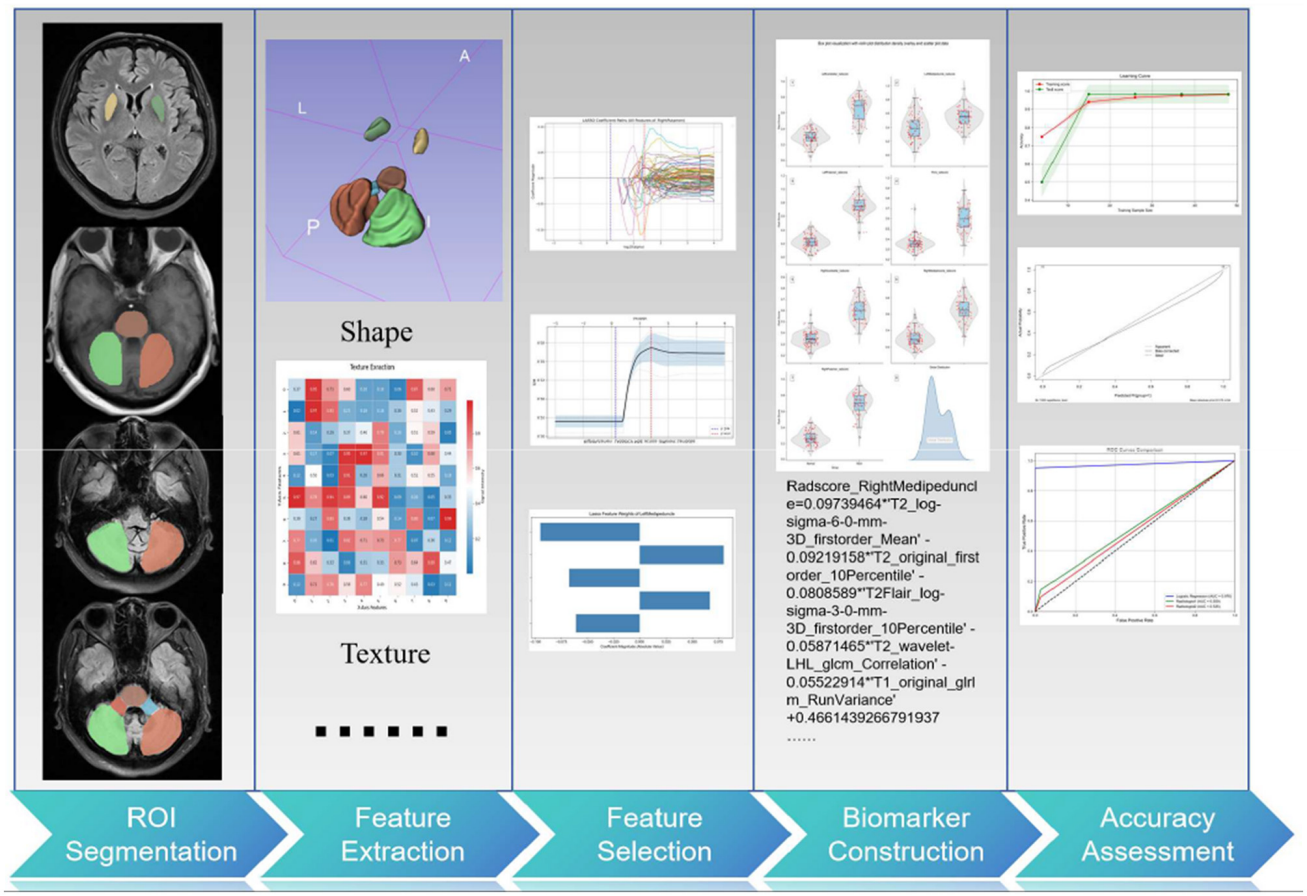
Technical workflow of this research.

The complete technical workflow is illustrated in Figure 1.

### Radiomics feature selection

To ensure robustness of radiomics features, 70% of randomly selected samples (*n* = 94/135) were allocated for feature extraction. Regions of interest (ROIs) were independently delineated by two certified radiologists (each with > 10 years of experience) following a standardized workflow described above, and features were extracted uniformly. Inter-observer agreement was evaluated using the intraclass correlation coefficient (ICC). Features demonstrating high reproducibility (ICC ≥ 0.75) were retained for subsequent analysis.

Retained features were Z-score normalization to eliminate scale differences, followed by application of the least absolute shrinkage and selection operator (LASSO) algorithm for region-specific feature selection. The optimal penalty coefficient (λ) was determined via 10-fold cross-validation (22), and the top five features with highest discriminative power per brain region were selected.

### Diagnostic model development and evaluation

The radiomics score (Rad-score) for each brain region was calculated as


(1) $$\begin{array}{l} \text{Rad}-\text{score}=\Sigma \left(\text{Feature Value}\times \text{Feature Weight}\right)+{\text{b}}_{0}, \end{array}$$

where Feature Weight denotes the coefficient derived from selected features, and *b*_0_ represents the intercept term.

For all 135 samples, region-specific Rad-scores were calculated to generated a multi-regional biomarker matrix comprising seven Rad-scores per subject. The dataset was split into training, testing, and validation cohorts in a 6:2:2 ratio (*n* = 81/27/27). A logistic regression (LR) model integrated the seven regional Rad-scores. In order to improve the stability of model evaluation, hierarchical 10-fold cross-validation is used on the training set (22), and the hyperparameters of each algorithm were optimized by grid search to determine the optimal parameter combination. The training curve was plotted on the training set to assess model performance. Classification reports were computed for both testing and validation cohorts.

The machine learning model was implemented using the scikit-learn Python library (version 1.5.1). The model performance was assessed using the area under the receiver operating characteristic curve (AUC) of the test set and the classification report, and the SHapley Additive exPlanations (SHAP) method was used to analyze the feature contribution and the decision logic of the model (23, 24). Finally, a nomogram was constructed to visualize the prediction results.

### Visual assessment of MRI scans

Two certified radiologists (each with >10 years of experience) independently performed a blinded assessment of 135 samples to evaluate suspicion of multiple system atrophy (MSA) diagnosis. This evaluation was based strictly on the MRI markers described in the 2022 International Movement Disorder Society (MDS) diagnostic criteria for MSA (6), without access to clinical information.

### Statistical analysis

Data analyses were performed using R software (v4.4.2) and Python (v3.9). Continuous variables conforming to a normal distribution were expressed as mean ± standard deviation (SD) and compared between groups using the independent samples *t*-test. Non-normally distributed data were presented as median (interquartile range) [M (IQR)] and analyzed via the Mann–Whitney *U* test. Categorical variables were reported as frequency (percentage) with intergroup comparisons conducted using Chi-square tests.

Machine learning model performance was evaluated using: AUC, Class-specific accuracy, recall, F1-score. Statistical differences in AUC values between machine learning models and interpretations by two radiologists were assessed using DeLong's test. Model interpretability was analyzed via the SHAP package (v0.43.0) in Python to quantify feature contributions. Inter-observer agreement of the visual judgments of MRI images between radiologists was evaluated using Cohen's kappa coefficient. A threshold of *P* < 0.05 was defined for statistical significance.

## Results

### Demographic characteristics

A total of 62 patients with clinically probable MSA (mean age 66.3 ± 7.8, 35 females), 73 healthy controls (mean age 67.6 ± 10.5, 31 females), and the same 62 MSA patients were enrolled. Statistical analysis showed that there was no significant difference in age between groups (Mann–Whitney *U* test, two-tailed test, *P* > 0.05), and there was no significant difference in gender distribution between groups (chi-square test, two-tailed test, *P* > 0.05). Detailed data on demographic characteristics are provided in Table 1.

**Table 1:** Demographic characteristics of study participants.

**Characteristic**	**Normal group (*n* = 73)**	**MSA group (*n* = 62)**	***P*-value**
**Gender**, ***n*** **(%)**			
Female	38 (52.1%)	24 (38.7%)	0.168^a^
Male	35 (47.9%)	38 (61.3%)	
**Age (years)**			
Median [Min, Max]	65.0 [38.0, 88.0]	67.0 [47.0, 83.0]	0.575^b^
IQR [Q1, Q3]	23.0 [54.0, 77.0]	15.0 [55.0, 70.0]	

a Gender differences were analyzed using the Chi-square test;

b age data violated the normality assumption (Shapiro–Wilk test: *P* = 0.0291 for Normal, *P* = 0.0304 for MSA group); therefore, intergroup age comparisons were performed with the Mann–Whitney *U* test.

### Feature selection and construction of Rad-score

Robust features demonstrating intraclass correlation coefficients (ICC) ≥ 0.75 were selected from multimodal composite features within each brain region. These features were subsequently subjected to least absolute shrinkage and selection operator (LASSO) regression analysis with 10-fold cross-validation. The five features exhibiting the strongest predictive weights (Supplementary Figure 1) were retained to construct the radiomics biomarker (Rad-score) using the formulas in Supplementary Table 2.

**Figure 2:**
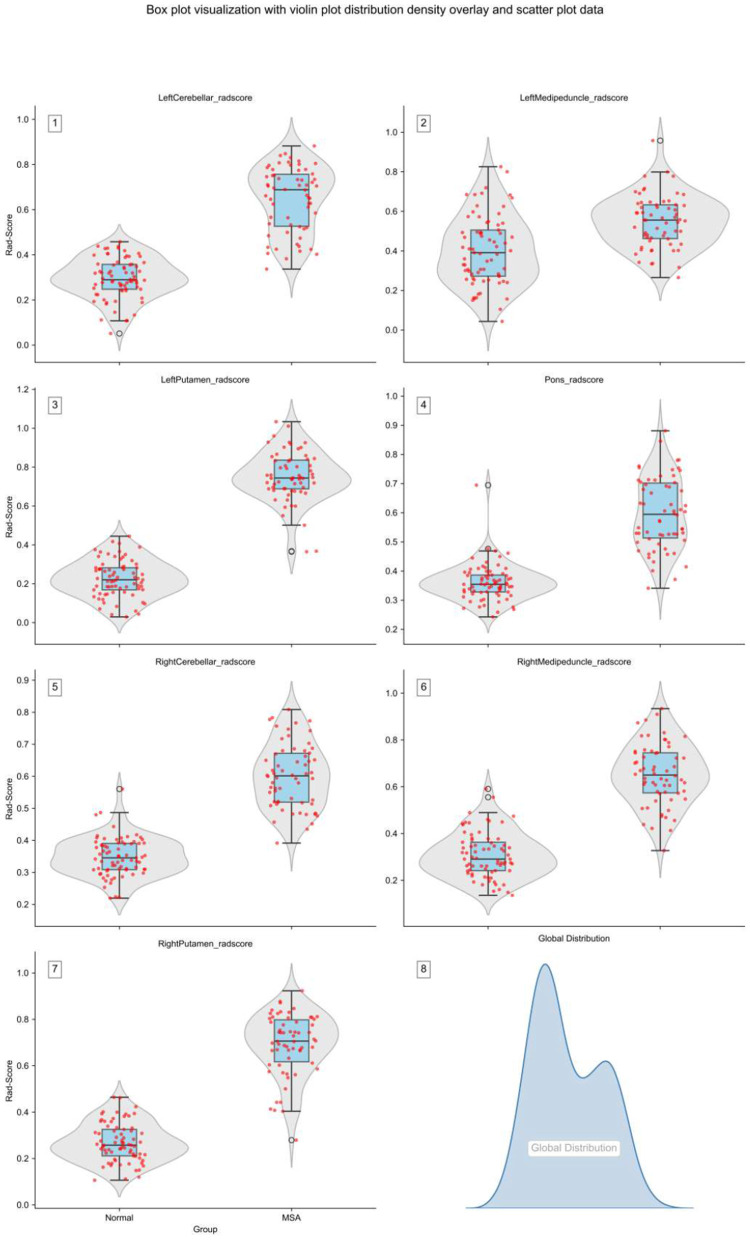
Regional distribution of Rad-scores across brain regions. The horizontal axis indicating the group categories and the vertical axis displaying the specific Rad-score values.

The radiomics signature (Rad-score) for each brain region was calculated using the aforementioned formula. Composite distribution plots of Rad-scores were subsequently generated (Figure 2). Intergroup differences were observed between the MSA cohort and healthy controls, indicating distinct distribution patterns.

Composite plot showing Rad-score distributions in specific brain regions. Figures 2.1–2.7 represent the left cerebellar, left medipeduncle, left putamen, pons, right cerebellar, right medipeduncle, and right putamen, respectively. Figure 2.8 displays the overall Rad-score distribution, with bimodal peaks indicating distinct mean values between groups.

### The LR model

The LR model was identified as the optimal predictive model and underwent further evaluation. The logistic equation is as follows:


$$\begin{array}{r} \text{log }\left(\text{P}/\left(1-\text{P}\right)\right)=\left(1.4005^{*}\text{ LeftCerebellar\_RADscore}\right)\\ +\left(0.5226^{*}\text{LeftMedipeduncle\_RADscore}\right)\\ +\left(2.0314^{*}\text{LeftPutamen\_RADscore}\right)\\ +\left(0.7332^{*}\text{Pons\_RADscore}\right)\\ +\left(0.9171^{*}\text{RightCerebellar\_RADscore}\right)\\ +\left(1.4922^{*}\text{RightMedipeduncle\_RADscore}\right)\\ +\left(1.6966^{*}\text{RightPutamen\_RADscore}\right)\\ -4.2657 \end{array}$$

**Figure 3:**
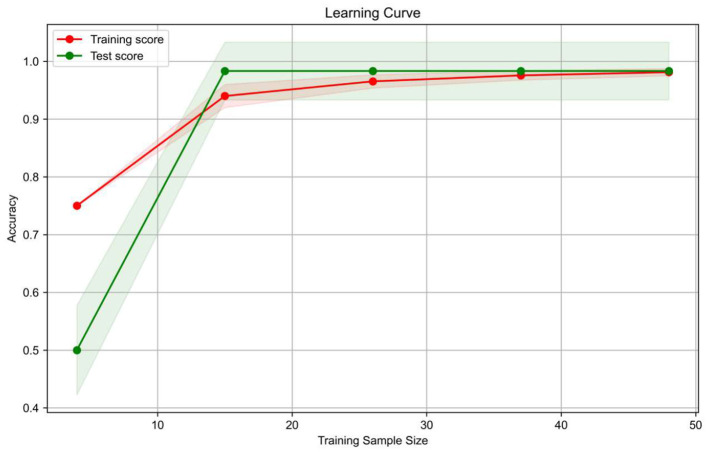
Illustrates the learning curve on the training cohort. As revealed by the learning curve, once the sample size surpasses 15, the test accuracy overtakes the training accuracy and steadily converges to 0.98 with further increases in sample size.

As evidenced by the learning curve derived from the training cohort (Figure 3), both training and test scores of the logistic regression (LR) model converged asymptotically toward 0.98. This convergence indicates the absence of overfitting and confirms robust generalization capabilities.

Performance metrics for the LR model across training, test, and validation sets are summarized in Table 2. The model's discriminative power and generalization characteristics for the two sample classes were comprehensively evaluated using four core metrics: Precision, Recall, F1-Score, and Support. All datasets exhibited high classification performance (Macro Avg F1 ≥ 0.95), establishing model robustness. The near-identical accuracies of the training set (Accuracy = 0.98) and test set (Accuracy = 0.97) further substantiate the absence of overfitting. In the validation set, moderately reduced recall (0.89) was observed for multiple system atrophy (MSA) samples relative to other datasets. Conversely, normal group samples achieved perfect recall (1.00) universally, demonstrating complete capture of this class. Notably, MSA classification consistently yielded precision of 1.00, indicating zero false positives.

**Table 2:** Classification report of the logistic regression model across training, test, and validation cohorts.

**Dataset**	**Class/statistic**	**Precision**	**Recall**	**F1-score**	**Support**	**Accuracy**
**Training**	**Normal**	0.96	1.00	0.98	27	–
	**MSA**	1.00	0.96	0.98	27	–
	Macro avg	0.98	0.98	0.98	–	–
	Weighted avg	0.98	0.98	0.98	54	–
	Overall	–	–	–	–	0.98
**Testing**	**Normal**	0.96	1.00	0.98	24	–
	**MSA**	1.00	0.94	0.97	16	–
	Macro avg	0.98	0.97	0.97	–	–
	weighted avg	0.98	0.97	0.97	40	–
	Overall	–	–	–	–	0.97
**Validation**	**Normal**	0.92	1.00	0.96	22	–
	**MSA**	1.00	0.89	0.94	19	–
	Macro avg	0.96	0.95	0.95	–	–
	Weighted avg	0.96	0.95	0.95	41	–
	Overall	–	–	–	–	0.95

### SHAP-based model interpretability analysis

**Figure 4:**
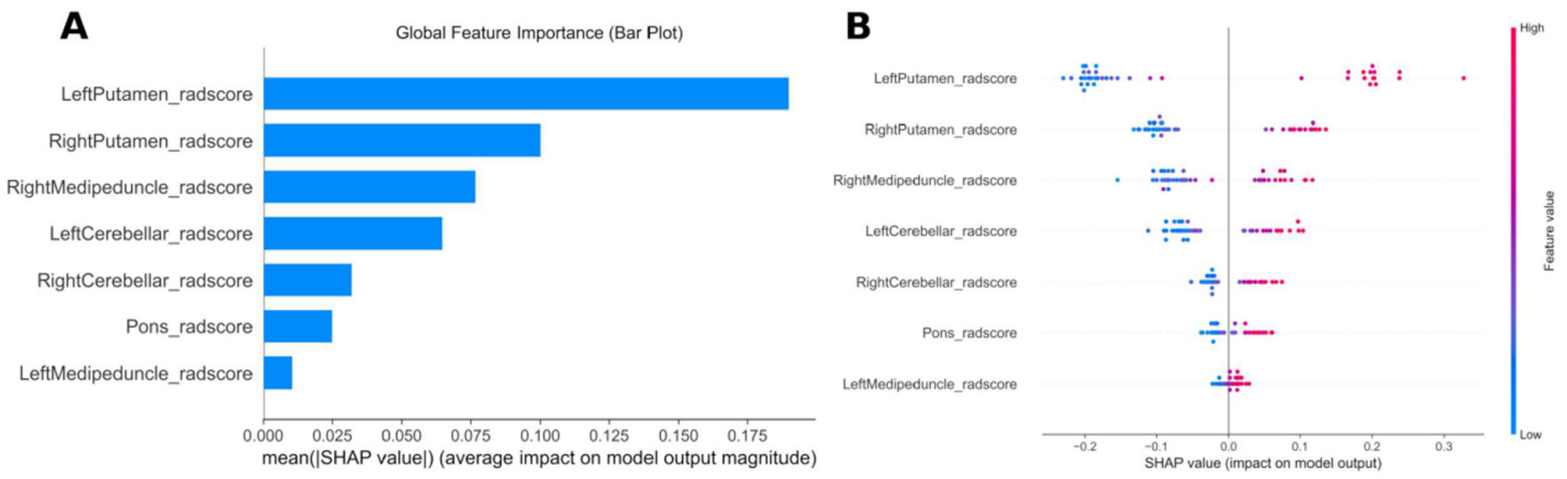
Interpretability analysis of LR models. **(A)** Importance ranking plot of features in the LR model. **(B)** SHAP dendrogram showing feature importance, correlations, and distributions in the LR model.

SHAP analysis was performed to interpret the contribution of regional radiomics signatures (RADscore) and the model's decision-making mechanism. Figure 4A illustrates the hierarchical feature importance in the prediction model, where the vertical axis ranks features by descending importance and the horizontal axis denotes the mean absolute SHAP value. The analysis identified the left putamen rad-score as the most influential predictor. Figure 4B provides a detailed summary plot of this ranking: each point represents an individual sample, with a color gradient (blue to red) indicating low-to-high feature magnitudes. The vertical axis sorts features by importance, while the distribution illustrates correlations between feature values and their corresponding SHAP values. SHAP analysis revealed significant lateralized contributions of imaging biomarkers across these brain regions.

### Construction of nomogram

**Figure 5:**
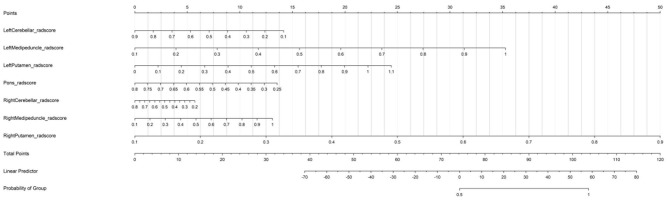
Nomogram for predicting the probability of multiple system atrophy (MSA).

Based on the established logistic regression model, a nomogram (Figure 5) predicting the probability of multiple system atrophy (MSA) was constructed using the following predictors: rad-scores of the left cerebellar hemisphere, left medipeduncle, left putamen, pons, right cerebellar hemisphere, right medipeduncle, and right putamen.

### Visual assessment of radiologists

**Figure 6:**
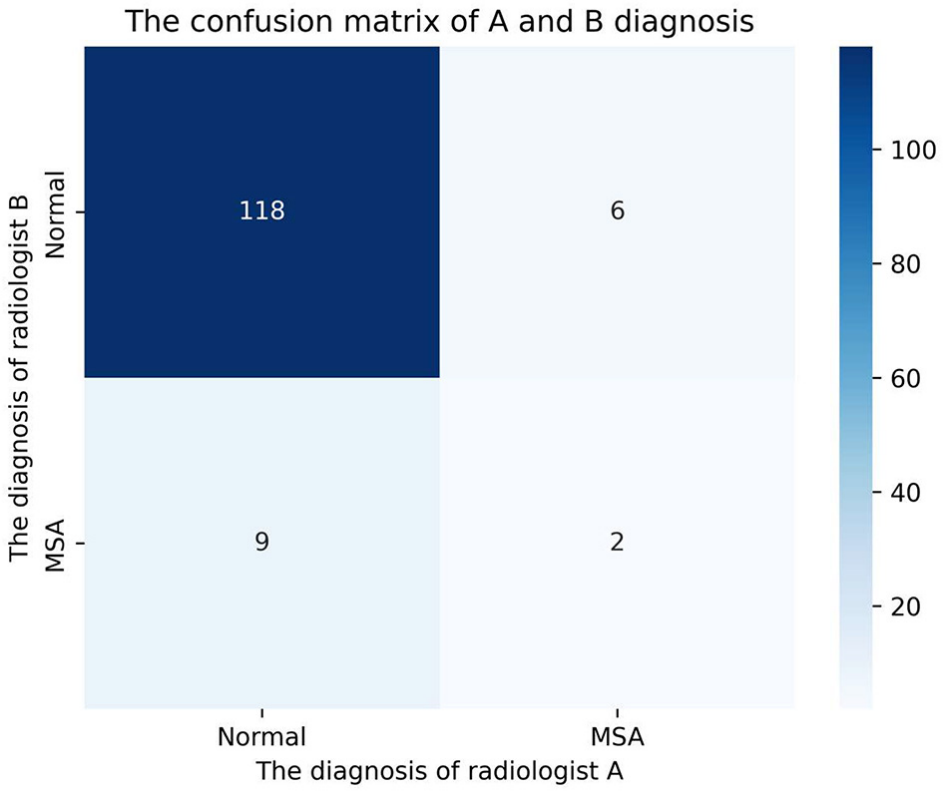
Confusion matrices of diagnostic assessments by two radiologists.

**Figure 7:**
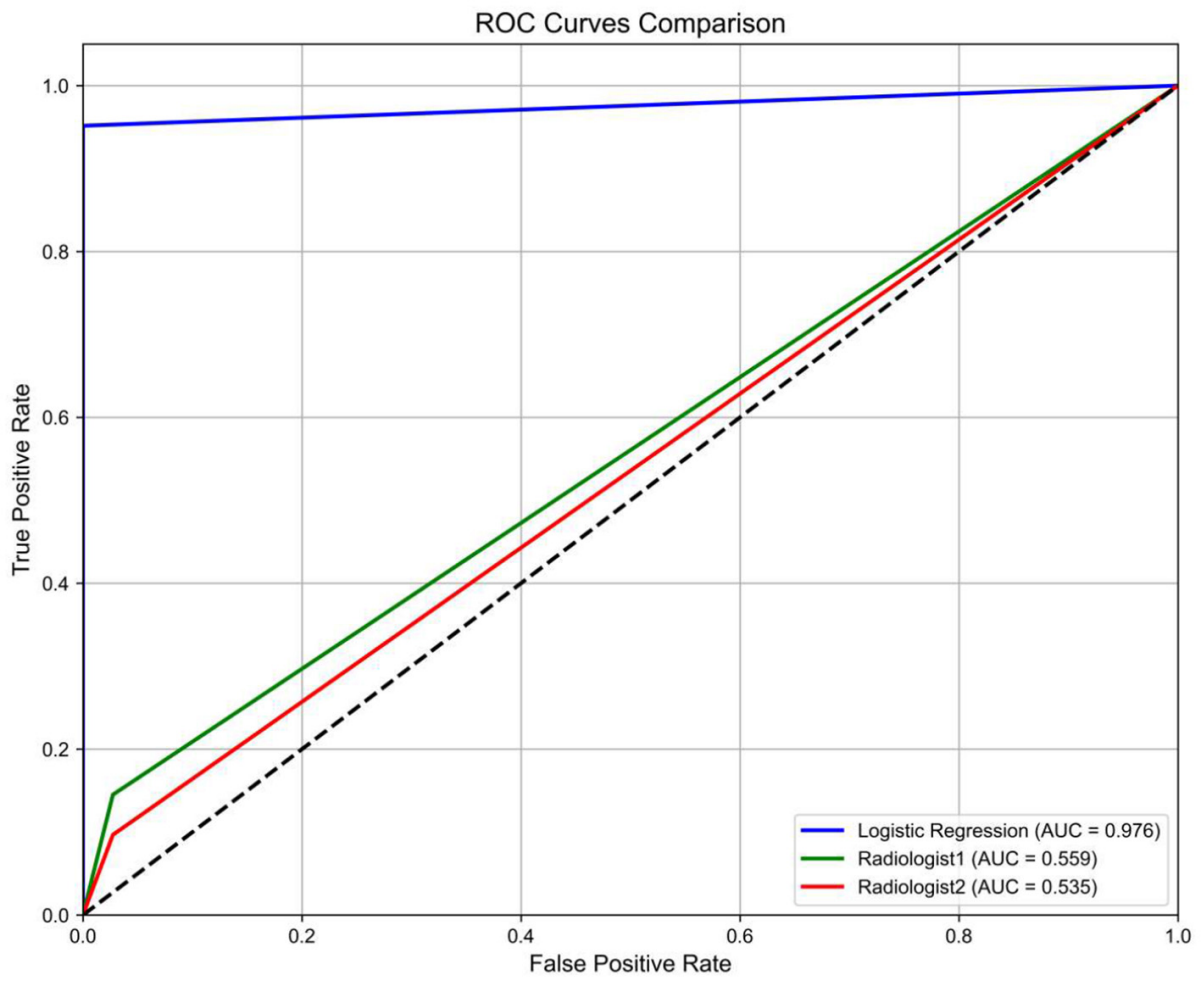
Diagnostic performance comparison. ROC curves demonstrate superior AUC of the LR model (0.976) vs. radiologists (A: 0.559, B: 0.535). Dashed line indicates random chance (AUC = 0.5).

Two radiologists performed independent assessments on 135 cases blinded to clinical information. Radiologist A classified 127 cases as normal and 8 as multiple system atrophy (MSA), while Radiologist B classified 124 as normal and 11 as MSA. Consensus diagnoses identified 118 normal cases and 2 MSA cases (Figure 6). The Cohen's kappa coefficient for inter-rater agreement was 0.152. Receiver operating characteristic (ROC) curves for the logistic regression (LR) model and both radiologists are shown in Figure 7, with areas under the curve (AUC) of 0.559 (95% CI: 0.48–0.63) for Radiologist A and 0.535 (95% CI: 0.45–0.62) for Radiologist B. The DeLong test comparing diagnostic performance between Radiologist A and Radiologist B yielded no significant difference (*Z* = 0.803, *P* = 0.422).

## Discussion

Multiple system atrophy (MSA) is a rare disease; because samples are hard to collect, studies are often characterized by small sample sizes and high-dimensional feature spaces, which can easily lead to overfitting if not handled properly. In this study, 70% of the samples were randomly selected for feature extraction of brain regions, aiming to reduce the excessive dependence of the model on the training data, thereby reducing the risk of overfitting. The core logic of this approach, a subsampling approach, is to use a random masking mechanism similar to Dropout to form regularization to improve generalization through data-level randomness (25–27). Previous studies have shown the utility of feature screening in small, high-dimensional data such as the one used in our study (28).

To efficiently identify the most discriminative features from extensive feature pools, we performed Z-score normalization on intraclass correlation coefficient (ICC)-validated features per brain region (29), then integrated four sequences (T1, T2, T2 FLAIR, ADC) into multimodal representations (30). Subsequently, LASSO regression was applied to extract key features from each region's multimodal set (31). Least absolute shrinkage and selection operator (LASSO) regression is a linear regression method combining feature selection and regularization. The core of LASSO regression is to realize sparse modeling by introducing L1 regularization term. Lasso regression can solve the problem of high-dimensional data redundancy by compressing the coefficients of unimportant features to zero and automatically screening key variables. Only a few nonzero coefficients are retained in the generated model, which improves the interpretation of the model. L1 regularization can deal with multicollinearity problems more effectively than ridge regression (L2 regularization) (32). Due to these characteristics, Lasso regression has been widely used in radiomics. Radiomics features are often in thousands of dimensions, and Lasso can simplify model parameters by filtering out 99% redundant features from the original features (33). Features were selected using internal 10-fold cross validation in the training set by the minimum mean squared error (MSE) (22). Among the non-zero weight features obtained from lasso regression, the five features with the greatest weight influence were selected as the variables to calculate the Rad-score. Based on the weight of feature variables and regression intercept construct of LASSO regression, Rad-score construction formulas (see Supplementary material) for seven brain regions were established as biomarker (34). The combined plot shows that the rad-score of each brain region has some discrimination power.

In order to maximize the model performance, a total of seven RAD-scores from seven brain regions in each sample were combined into a new research sample for logistic regression modeling. In order to improve the generalization ability, the total 135 samples were randomly divided into training group, test group and validation group according to 6:2:2 (35). The training group was used for modeling, and the learning curve within that group was plotted. The learning curve began to converge when the training sample reached 15, and the test score and training score increased with the training sample, and tended to converge to a curve pattern with the same value, indicating that the model did not overfit, showing that the model had good generalization ability (36). Among the key indicators, only MSA in the validation group achieved a recall rate of 0.89, while the others achieved an accuracy rate and recall rate of more than 0.9, showing excellent classification functions on the validation set and the test cohorts (37). This 0.89 sensitivity highlights the model's potential as a screening tool for MSA.

In this study, we demonstrated that the radiomics-based Rad-score exhibited greater sensitivity than conventional MRI imaging markers currently incorporated in diagnostic criteria for multiple system atrophy (MSA). The seven brain regions delineated in this study are the MRI imaging biomarkers mentioned in the diagnostic criteria of MSA, and can serve as on basis for clinical diagnosis of MSA (6). Although MRI abnormalities in MSA patients have high specificity, their sensitivity is usually low. Moreover, the clinical utility of these MRI findings in improving diagnostic accuracy remains to be fully elucidated (38). All cases included in this study were diagnosed as clinically probable multiple system atrophy (MSA) due to the absence of characteristic MRI findings. For further validation, 135 samples were evaluated by two radiologists with more than 10 years of experience. The AUCs for Radiologist A and Radioligist B were 0.559 and 0.535, and the kappa coefficient of agreement between them was 0.152. These results demonstrate that macroscopic MRI features alone were insufficient for accurate diagnosis in this study. In contrast, the diagnostic model based on the Rad-score derived from seven brain regions showed excellent classification performance, supporting its practical utility.

The weights of the RADscores of these seven brain regions in the model found in this study are also consistent with the laterality characteristics of MSA found by other research methods. The SNAP diagram of the Logistic regression model shows that among the seven brain regions, the influence weights are ranked as follows: left putamen > right putamen > right medipeduncle > left cerebellar > right cerebellar > pons > left medipeduncle. The results showed that the influence of the left putamen was greater than that of the right medipeduncle, and the influence of the left cerebellar hemisphere was greater than that of the right cerebellar hemisphere. These findings may be related to the pathogenesis of MSA. There is also an important tendency of hemisphere lateralization in the process of PD. Therefore, PD is considered an inherently asymmetric disease in clinical practice. This clinical asymmetry is associated with more severe contralateral nigrostriatal degeneration (39). Some studies have shown a “left hemisphere susceptibility” in this condition, as the left nigrostriatal pathway is more affected than the right (40). Previous PET imaging studies based on altered ^18^F-DOPA uptake have confirmed that the loss of ^18^F-DOPA uptake rate in the nigrostriatal system in selected populations of drug-naive Parkinson's disease cohorts is predominantly on the most affected side, so that the left hemisphere image depicts the more affected side. While the less affected side (LAS) corresponds to the right hemisphere, the reduced topography was mainly in the putamen of the left hemisphere with maximum uptake loss in the anterior-posterior axis and dorsoventral axis, respectively (41). In the study by Van Laere and colleagues, left putamen uptake was observed in 24 of 38 patients (63.1%) with right-sided predominant disease (*P* < 0.001), indicating that this laterality is also present in IPD such as MSA (42). The dopaminergic system is thought to be primarily responsible for this lateralization due to its critical role in motor control. Inherent interhemispheric imbalances in nigrostriatal dopamine (DA) levels in humans and animals have been shown to be associated with lateralization of motor behavior (43). This change can cause the corresponding changes in the images of the putamen. Although such changes cannot be detected in the macroscopic image features, the RADscore constructed by radiomics can accurately detect the changes in the left and right putamen. In the MSA group in the present study, the changes of the left putamen were greater than those of the right putamen, which is consistent with previous studies.

Minori Furuta et al. found that MSA patient exhibited laterality changes in the middle cerebellar peduncle on SPECT (44). While conventional MRI failed to reveal these alterations, radiomics captured them and confirmed laterality patterns reported previously. Similarly, Francesca Caso observed atrophy of the left cerebellar hemisphere but not the right cerebellar hemisphere in patients with MSA-P by 1.5T magnetic resonance imaging, suggesting that atrophy of the left cerebellar hemisphere may be more easily observed at the macroscopic level than that of the right (45). This study found that the effect of the left cerebellar hemisphere is also called right hemisphere enlargement, which is consistent with this. These results are consistent with the laterality of previous studies, and further support the proposed RADscore as a biomrker to not only preferentially screen out highly suspected MSA cases. In addition, Eun Hye Jeong et al. found through ^123^I-FP-CIT SPECT study that the asymmetry of putamen was more obvious in the early stage of the disease, and this asymmetry decreased with the extension of follow-up time (46). The patients in this study belonged to the early stage of the disease when none of the macroscopic imaging markers required by the guidelines were found, so the RADscore difference of the putamen was more significant. The Rad-score may serve as a potential biomarker for the early diagnosis of multiple system atrophy (MSA). The diagnostic model based on the Rad-score demonstrates promising diagnostic performance in identifying MSA cases.

## Conclusion

In conclusion, for patients with clinically suspected multiple system atrophy (MSA) but lacking definitive MRI markers, the radiomics-based RAD score offers a sensitive imaging biomarker that enables the construction of a diagnostic model capable of distinguishing MSA from healthy controls and improving overall diagnostic accuracy.

## Limitations

This study has several limitations. First, it was a single-center retrospective analysis, which may limit the generalizability of the findings to broader or more diverse populations. Second, although we included patients with clinically probable MSA and healthy controls, the diagnosis was primarily based on clinical criteria, which may introduce selection bias. Third, the radiomics model was built using manually delineated regions of interest (ROIs), and thus may be subject to inter- and intra-observer variability; future studies incorporating automated segmentation techniques are warranted. Finally, external validation using an independent cohort is needed to further confirm the robustness and clinical applicability of the RAD score as a diagnostic biomarker.

## Data Availability

The original contributions presented in the study are included in the article/Supplementary material, further inquiries can be directed to the corresponding authors.
