# Supplementary material for: Development of a radiomics-based model for diagnosis of multiple system atrophy using multimodal MRI
Source: Front Neurol. 2025 Sep 8;16:1650350. doi: 10.3389/fneur.2025.1650350 (PMC12450663; doi:10.3389/fneur.2025.1650350)
Supplement: Supplementary file 1 [file Supplementary_file_1.docx]

**Supplementary**

**
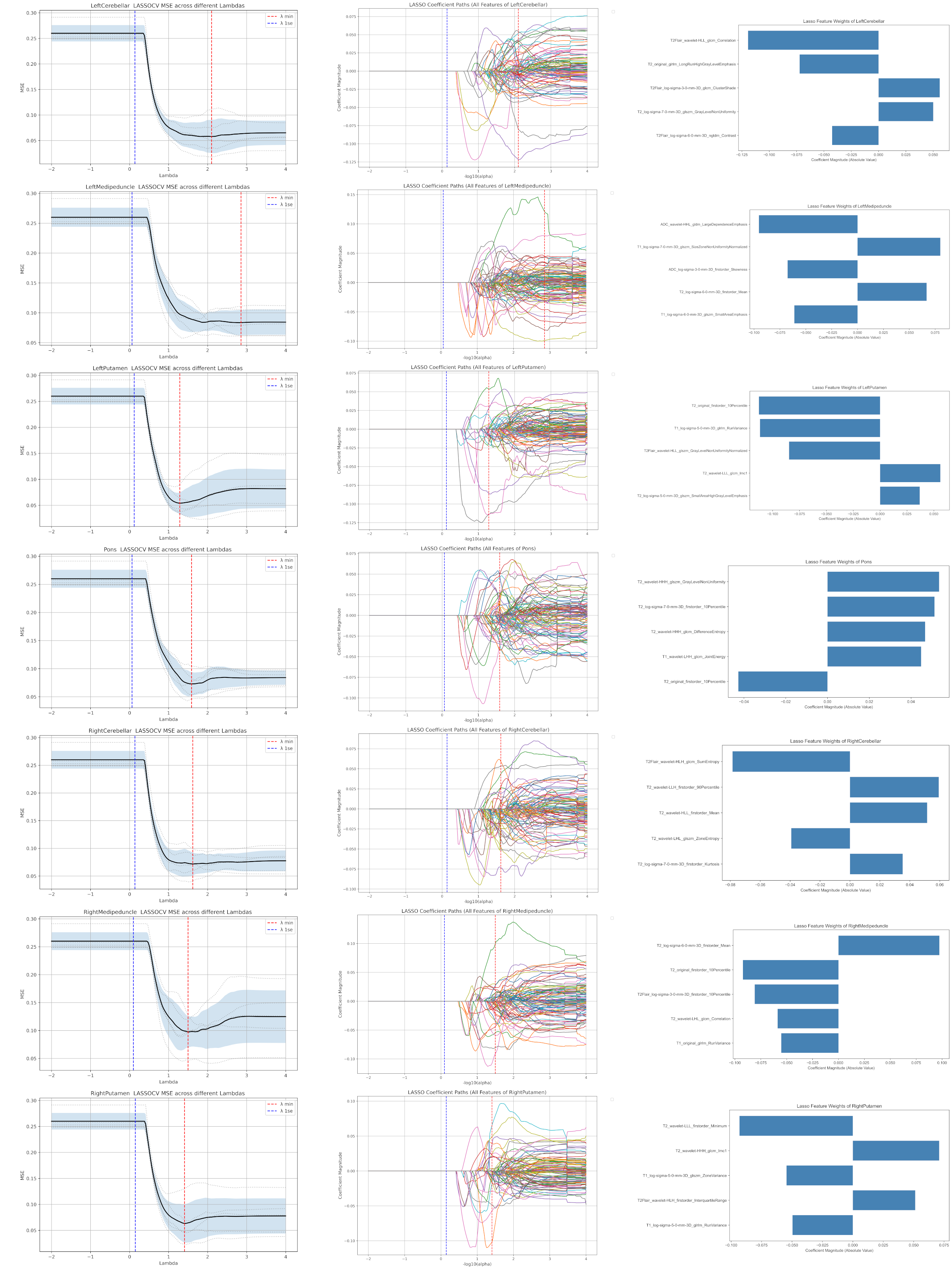
**

**SupFig. 1** Each row corresponds to one of the seven ROIs (top to bottom: left cerebellar hemisphere, left middle cerebellar peduncle, left putamen, pons, right cerebellar hemisphere, right middle cerebellar peduncle, right putamen).

Left panel: LASSO regularization path. λmin (red dashed line) denotes the cross-validated tuning parameter yielding the minimum deviance; λ1se (blue dashed line) denotes the largest λ within one standard error of λmin.

Middle panel: coefficient progression. Traces illustrate how each radiomic feature’s coefficient evolves with λ; features retaining non-zero coefficients at the selected λ are retained.

Right panel: feature weights. Bar plot displays the five most influential selected features and their normalized LASSO coefficients.

| **SupTable1 Features and Coefficients Selected by Lasso Regression​** | | |
| --- | --- | --- |
| ****Brain Region​**​** | **​**​Feature Name**** | **​**​Weight**** |
| ​****​Left Cerebellar​****​ | T2Flair_wavelet-HLL_glcm_Correlation | -0.11936 |
|  | T2_original_glrlm_LongRunHighGrayLevelEmphasis | -0.07220 |
|  | T2Flair_log-sigma-3-0-mm-3D_glcm_ClusterShade | 0.05593 |
|  | T2_log-sigma-7-0-mm-3D_glszm_GrayLevelNonUniformity | 0.05003 |
|  | T2Flair_log-sigma-6-0-mm-3D_ngtdm_Contrast | -0.04248 |
| ​****​Right Cerebellar​****​ | T2Flair_wavelet-HLH_glcm_SumEntropy | -0.07847 |
|  | T2_wavelet-LLH_firstorder_90Percentile | 0.05936 |
|  | T2_wavelet-HLL_firstorder_Mean | 0.05158 |
|  | T2_wavelet-LHL_glszm_ZoneEntropy | -0.03924 |
|  | T2_log-sigma-7-0-mm-3D_firstorder_Kurtosis | 0.03523 |
| ​****​Left Medipeduncle​****​ | ADC_wavelet-HHL_gldm_LargeDependenceEmphasis | -0.09528 |
|  | T1_log-sigma-7-0-mm-3D_glszm_SizeZoneNonUniformityNormalized | 0.08001 |
|  | ADC_log-sigma-3-0-mm-3D_firstorder_Skewness | -0.06759 |
|  | T2_log-sigma-6-0-mm-3D_firstorder_Mean | 0.06672 |
|  | T1_log-sigma-6-0-mm-3D_glszm_SmallAreaEmphasis | -0.06108 |
| ​****​Right Medipeduncle​****​ | T2_log-sigma-6-0-mm-3D_firstorder_Mean | 0.09739 |
|  | T2_original_firstorder_10Percentile | -0.09219 |
|  | T2Flair_log-sigma-3-0-mm-3D_firstorder_10Percentile | -0.08086 |
|  | T2_wavelet-LHL_glcm_Correlation | -0.05871 |
|  | T1_original_glrlm_RunVariance | -0.05523 |
| ​****​Left Putamen​****​ | T2_original_firstorder_10Percentile | -0.11271 |
|  | T1_log-sigma-5-0-mm-3D_glrlm_RunVariance | -0.11172 |
|  | T2Flair_wavelet-HLL_glszm_GrayLevelNonUniformityNormalized | -0.08471 |
|  | T2_wavelet-LLL_glcm_Imc1 | 0.05607 |
|  | T2_log-sigma-5-0-mm-3D_glszm_SmallAreaHighGrayLevelEmphasis | 0.03694 |
| ​****​Right Putamen​****​ | T2_wavelet-LLL_firstorder_Minimum | -0.09314 |
|  | T2_wavelet-HHH_glcm_Imc1 | 0.07101 |
|  | T1_log-sigma-5-0-mm-3D_glszm_ZoneVariance | -0.05453 |
|  | T2Flair_wavelet-HLH_firstorder_InterquartileRange | 0.05130 |
|  | T1_log-sigma-5-0-mm-3D_glrlm_RunVariance | -0.04956 |
| ​****​Pons​****​ | T2_wavelet-HHH_glszm_GrayLevelNonUniformity | 0.05344 |
|  | T2_log-sigma-7-0-mm-3D_firstorder_10Percentile | 0.05122 |
|  | T2_wavelet-HHH_glcm_DifferenceEntropy | 0.04673 |
|  | T1_wavelet-LHH_glcm_JointEnergy | 0.04483 |
|  | T2_original_firstorder_10Percentile | -0.04270 |

Column 1: Region of interest (ROI); Column 2: Five most discriminative radiomic features per ROI; Column 3: Normalized LASSO coefficient (feature weight).

| **SupTable2 RadScore Calculation Formula for Brain Regions** | |
| --- | --- |
| ****Radscore of Brain Region​**​** | **​**​Calculation Formula**** |
| ****​Radscore of Left Cerebellar​****​ | -0.11935983*'T2Flair_wavelet-HLL_glcm_Correlation'  -0.07219744*'T2_original_glrlm_LongRunHighGrayLevelEmphasis'  +0.05593435*'T2Flair_log-sigma-3-0-mm-3D_glcm_ClusterShade'  +0.05002845*'T2_log-sigma-7-0-mm-3D_glszm_GrayLevelNonUniformity'  -0.04247527*'T2Flair_log-sigma-6-0-mm-3D_ngtdm_Contrast'  +0.45655525788640217 |
| ​****​Radscore of Right Cerebellar​****​ | -0.07846936*'T2Flair_wavelet-HLH_glcm_SumEntropy'  +0.05936014*'T2_wavelet-LLH_firstorder_90Percentile'  +0.05158274*'T2_wavelet-HLL_firstorder_Mean'  -0.03924128*'T2_wavelet-LHL_glszm_ZoneEntropy'  +0.0352259*'T2_log-sigma-7-0-mm-3D_firstorder_Kurtosis'  +0.4645789459098546 |
| ​****​Radscore of Left Medipeduncle​****​ | -0.09527955*'ADC_wavelet-HHL_gldm_LargeDependenceEmphasis'  +,0.0800061*'T1_log-sigma-7-0-mm-3D_glszm_SizeZoneNonUniformityNormalized'  -0.06758936*'ADC_log-sigma-3-0-mm-3D_firstorder_Skewness'  +0.06672209*'T2_log-sigma-6-0-mm-3D_firstorder_Mean'  -0.06108168*'T1_log-sigma-6-0-mm-3D_glszm_SmallAreaEmphasis'  +0.47026464984298083 |
| ​****​Radscore of Right Medipeduncle​****​ | 0.09739464*'T2_log-sigma-6-0-mm- 3D_firstorder_Mean'  -0.09219158*'T2_original_firstorder_10Percentile'  -0.0808589*'T2Flair_log-sigma-3-0-mm-3D_firstorder_10Percentile'  -0.05871465*'T2_wavelet-LHL_glcm_Correlation'  -0.05522914*'T1_original_glrlm_RunVariance'  +0.4661439266791937 |
| ​****​Radscore of Left Putamen​****​ | -0.11271407*'T2_original_firstorder_10Percentile'  -0.1117247*'T1_log-sigma-5-0-mm-3D_glrlm_RunVariance'  -0.08471044*'T2Flair_wavelet-HLL_glszm_GrayLevelNonUniformityNormalized'  +0.05607094*'T2_wavelet-LLL_glcm_Imc1'  +0.03693519*'T2_log-sigma-5-0-mm-3D_glszm_SmallAreaHighGrayLevelEmphasis'  +0.46707095118527636 |
| ​****​Radscore of Right Putamen​****​ | -0.09314264*'T2_wavelet-LLL_firstorder_Minimum'  +0.07100788*'T2_wavelet-HHH_glcm_Imc1'  -0.0545264*'T1_log-sigma-5-0-mm-3D_glszm_ZoneVariance'  +0.05130301*'T2Flair_wavelet-HLH_firstorder_InterquartileRange'  -0.04956479*'T1_log-sigma-5-0-mm-3D_glrlm_RunVariance'  +0.46282302131857544 |
| ​****​Radscore of Pons​****​ | 0.05343895*'T2_wavelet-HHH_glszm_GrayLevelNonUniformity'  +0.05121945*'T2_log-sigma-7-0-mm-3D_firstorder_10Percentile'  +0.04672809*'T2_wavelet-HHH_glcm_DifferenceEntropy'  +0.04482831*'T1_wavelet-LHH_glcm_JointEnergy'  -0.0426962*'T2_original_firstorder_10Percentile'  +0.4691384950946552 |
